# The role of antibody-dependent enhancement in dengue vaccination

**DOI:** 10.1186/s40794-024-00231-2

**Published:** 2024-11-01

**Authors:** D. G. Aynekulu Mersha, I. van der Sterren, L. P.M. van Leeuwen, T. Langerak, M. S. Hakim, B. Martina, S. F.L. van Lelyveld, E. C.M van Gorp

**Affiliations:** 1 Department of Viroscience, Erasmus Medical Center, Dr. Molewaterplein 40, PO Box Ee-1722, Rotterdam, 3015 GD the Netherlands; 2 Postgraduate School of Molecular Medicine, Erasmus Medical Center, Rotterdam, the Netherlands; 3 Artemis Bioservices and Athenavax B.V, Delft, the Netherlands; 4 Department of internal medicine, Spaarne Gasthuis, Haarlem/Hoofddorp, the Netherlands

## Abstract

Dengue is the most rapidly spreading vector-borne disease worldwide, with over half the global population at risk for an infection. Antibody-dependent enhancement (ADE) is associated with increased disease severity and may also be attributable to the deterioration of disease in vaccinated people. Two dengue vaccines are approved momentarily, with more in development. The increasing use of vaccines against dengue, combined with the development of more, makes a thorough understanding of the processes behind ADE more important than ever. Above that, due to the lack of treatment options, this method of prevention is of great importance. This review aims to explore the impact of ADE in dengue vaccinations, with the goal of enhancing potential vaccination strategies in the fight against dengue.

## Introduction

Dengue is the most rapidly spreading vector-borne disease worldwide, with over half the global population at risk for infection [1, 2]. An important factor associated with increased severity of a dengue infection is antibody-dependent enhancement (ADE) [3]. ADE may also cause increased disease severity in vaccinated people [4]. Currently, two vaccines against dengue are approved, with more in development, making a good understanding of the processes behind ADE more vital than ever [5].

ADE is an immune-pathological phenomenon associated with increased disease severity in multiple viral infections [3, 6]. This process is mediated by cross-reactive, non-neutralizing antibodies or antibodies in non-neutralizing concentrations. Viral infections where ADE is seen include several Flaviviruses, Coronaviruses, Ebola, HIV, RSV, measles and influenza, with dengue virus (DENV) being the most prominent example [6–16]. DENV is an arthropod-borne virus belonging to the family of the Flaviviridae, transmitted by mosquitos from the *Aedes* genus, such as *Aedes aegypti* and *Aedes albopictus*. These mosquitos are found in tropical and subtropical areas, mostly in Asia, Africa and South America, but have also been seen in colder areas such as France, the continental United States and the south of Brazil [17–20]. The incidence of DENV continues to increase, with an 85% rise in dengue cases between 1990 and 2019 [21]. Recent estimates of the incidence are between 50 and 100 million symptomatic infections per year and up to 390 million infections in total [22–24]. Of these patients, approximately 14.000 succumb to the disease annually [25]. An important factor in the increasing incidence of dengue infections is climate change. The rising global temperatures contribute to the proliferation of *Aedes* mosquitoes, extending their habitat and consequently widening the regions with potential dengue outbreaks [26, 27]. Global warming not only expands the habitat for the disease vector, but also decreases the extrinsic incubation time of dengue virus [28]. Other factors contributing to the increased incidence of dengue are viral evolution, socioeconomic factors and globalization [29].

DENV consists of four widely distributed serotypes, denoted as DENV1 through DENV4. In the context of dengue infection, individuals exposed to a particular serotype develop lifelong immunity against that specific serotype, however, immunity against the remaining serotypes is short-lived [30]. After a brief period of heterotypical immunity, subsequent infection with a different serotype poses an increased risk of more severe disease due to ADE [3, 31]. In regions where multiple serotypes coexist, co-infections involving multiple dengue serotypes at the same time may occur [32, 33].

The onset of dengue symptoms typically occurs 5 to 7 days following infection and manifests in three distinct phases. The febrile phase starts with the sudden onset of high fever, accompanied by a rash, as well as head and body aches. Subsequently, the critical phase ensues, characterised by plasma leakage and a reduction in blood platelet count. The final stage is the recovery phase, during which extravascular fluid is reabsorbed [34, 35]. It is noteworthy that a majority of patients recover after the febrile phase and do not progress to the critical phase. Clinically, dengue infections are categorized based on symptoms, aligning with the 2009 World Health Organization (WHO) guidelines as either dengue without or dengue with warning signs and severe dengue (SD) [34]. However, it is pertinent to acknowledge that the 1997 WHO guideline remains widely used, classifying infections as dengue fever, dengue haemorrhagic fever and dengue shock syndrome [36].

Treatment of dengue is mostly symptomatic, with antipyretics and fluid resuscitation being the cornerstone [37]. Until now, no antiviral drug has been officially approved for the treatment of dengue [38]. Due to the lack of treatment options, prevention is of great importance. However, increased disease severity has been seen after vaccination, which may be attributed to ADE. Therefore, the requirement rises for the development of a tetravalent vaccine that does not enhance a naturally derived infection. This necessity embodies a grand challenge. This review aims to explore the impact of ADE in dengue vaccinations, with the goal of enhancing potential vaccination strategies in the fight against dengue.

### ADE

ADE is a phenomenon that causes increased disease severity in viral infections. Various mechanisms underlie ADE, categorizing them into two primary groups: extrinsic ADE and intrinsic ADE. Extrinsic ADE causes increased entry of viral particles into immune cells, whereas intrinsic ADE describes the effects of this virus-immune complexes for modulation of the immune response, resulting in a “virus-friendly” intracellular environment where replication could be enhanced [39].

The most prominent example of ADE is seen in DENV. DENV infections can cause both extrinsic and intrinsic ADE. The root cause behind DENV induced ADE is starting with different dengue serotypes causing cross-reactive antibodies, where infection with one serotype grants only limited protection against other serotypes [30]. These heterotypic antibodies can bind to dengue virions of different serotypes, but do not neutralise the virus. Instead, these could cause enhanced inflammation and viral entry into immune cells, where the virus can multiply [3]. This also explains why the vast majority of SD cases are secondary infections, yet only a minority of these secondary infections develop SD [40].

When looking at populations at risks, children with a low antibody titre had a hazard ratio of 1,75 of developing dengue with warning signs or SD compared to DENV-naïve children [3]. Another group at risk for ADE is infants of mothers with pre-existing dengue antibodies. During pregnancy IgG antibodies cross the placenta to the foetal circulation, however, these antibodies decrease to sub-neutralising levels a few months after birth, before disappearing completely [41]. These sub-neutralising antibody levels are accompanied by the risk of ADE. This risk is the highest 6–9 months after birth, with a relative risk of 4 compared to infants of 12 months old [41].

#### Extrinsic ADE

When looking at mechanisms of extrinsic ADE, a first mechanism is fragment crystallisable gamma receptors (FcγR) mediated ADE, which uses the Fc-portion of an immunoglobulin (Ig) and the Fc-receptor on immune cells for increasing viral entry. Particularly the FcγR is the adhesion site, which is present as multiple differing subtypes on cells of both the innate and adaptive immune system [42].Poorly-neutralising Igs or Igs in sub-neutralising concentrations bind to viral surface proteins. These immunocomplexes bind to FcγR and augment the efficacy of the phagocytic pathway to gain entry to the cell [7, 43]. By enhancing internalization, viral load augments and starts a vicious cycle in which more cells are target of virus internalization and intracellularly DENV starts suppressing innate immune signalling [42]. Type I FcγR are present in three types with type II and type III further divided into different subtypes. These can bind only IgG, whereas IgA and IgE can bind to FcαR and FcεR respectively. These receptors can be grossly categorized as predominantly activating or inhibitory and are widely expressed on both lymphoid and myeloid cells. Each cell type has a distinctive distribution of FcγRs with mostly both activating and inhibitory receptors present on the cell surface. B- and natural killer (NK) cells are an exception to this adagio with B cells only expressing FcγRIIb, while NK cells exclusively express the activating receptor FcγRIIIa [44]. Examples of Fc-receptor mediated activating functions are inducement of cytokine production and inducing the release of granules produced by NK cells known as antibody-dependent cellular cytotoxicity. FcγR IIb is the inhibitory receptor regulating the broad spectrum of activating effector functions [42]. The process of binding Igs is regulated dynamically, with cell surface expression being modulated by cytokines in a way that pro-inflammatory cytokines upturn expression of activating FcγRs over FcγRIIb, the inhibitory counterpart. On the other hand, anti-inflammatory cytokines downregulate activating FcγRs and augment FcγRIIb [44]. The most represented Ig subtypes in blood are IgG1 and IgG2, both having a different preference for the Fc-receptors [45]. This dynamic process of regulating binding affinity is mostly regulated by core modulation of the Fc part of an Ig, thereby regulating the binding activity and affinity to his receptor. One of the key mechanisms is by fucosylation. Fc parts of an Ig without a core containing fucose are much more affinate to the activating FcγRIIIa. In patients with SD, afucosylated IgG1 binding FcγRIIIa, incapable of neutralizing dengue related antigens is overrepresented [43]. This could possibly make it a diagnostic tool [46].

This process of cell entry could be either with or without the complement cascade playing a role. The complement cascade comprises a cascade of nine factors, containing multiple proteins colliding in a vast consecutive matter which causes enhanced upregulation of the innate immune system, opsonisation of antigens and the lysis of pathogens [47, 48]. A part of the first factor of the complement system plays a role in ADE. When antigens are bound by the Fc- part of an Ig, complement factor 1q (C1q) is able to bind this antigen-antibody complex and facilitate binding to the FcγR of a host-cell [49]. When C1q gets involved, it keeps remaining a host-protective function against the amelioration of ADE. There are multiple hypothesis on the exact mechanisms of protection. The first is the improvement of the binding affinity of Fc-parts of an Ig. By improvement of the binding affinity or lowering of the stoichiometric threshold, the number of antibodies that must bind to an antigen is lower to counterbalance infectivity. The possibly lower amount of Igs needed to be effective could also be accounted to the steric interference of C1q on the Fcγ- receptors. Except for FcγRI, all Fc-receptors have low affinity to monomeric IgG’s. Only multimeric IgG’s are able to bind to Fc-receptors. C1q reduces the number of antibodies that must bind the virion to achieve neutralizing activity. This formation of antigen-antibody-complement complexes, eliciting the release of pro-inflammatory cytokines, might be dependent on several factors, including the specificity of the Ig and the type of FcγR. The second theory is that the pH-induced conformational changes, necessary for DENV to be infectious are restricted by C1q [43, 50–52].

Not only IgG, but also IgA and IgE were seen as possible facilitators of ADE. This was implicated in HIV related research where virus entry was seen into monocytes. Yet, in dengue in vitro research showed either a protective role for IgA, stimulating a much lower cytokine release after cell entry and facilitating a much less efficient internalization of DENV [53].

#### Intrinsic ADE

**Fig. 1:**
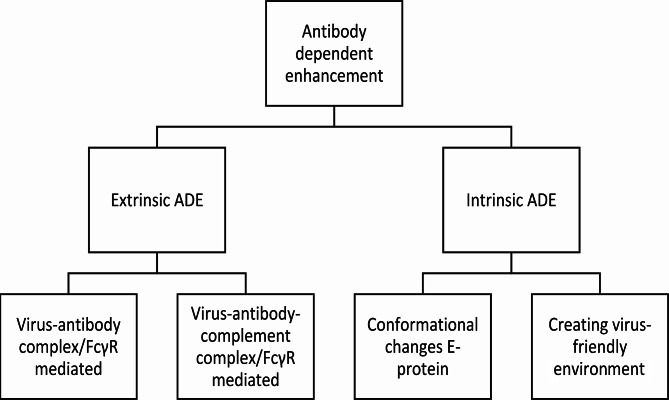
Organizational chart showing the different mechanisms of antibody-dependent enhancement

**Fig. 2:**
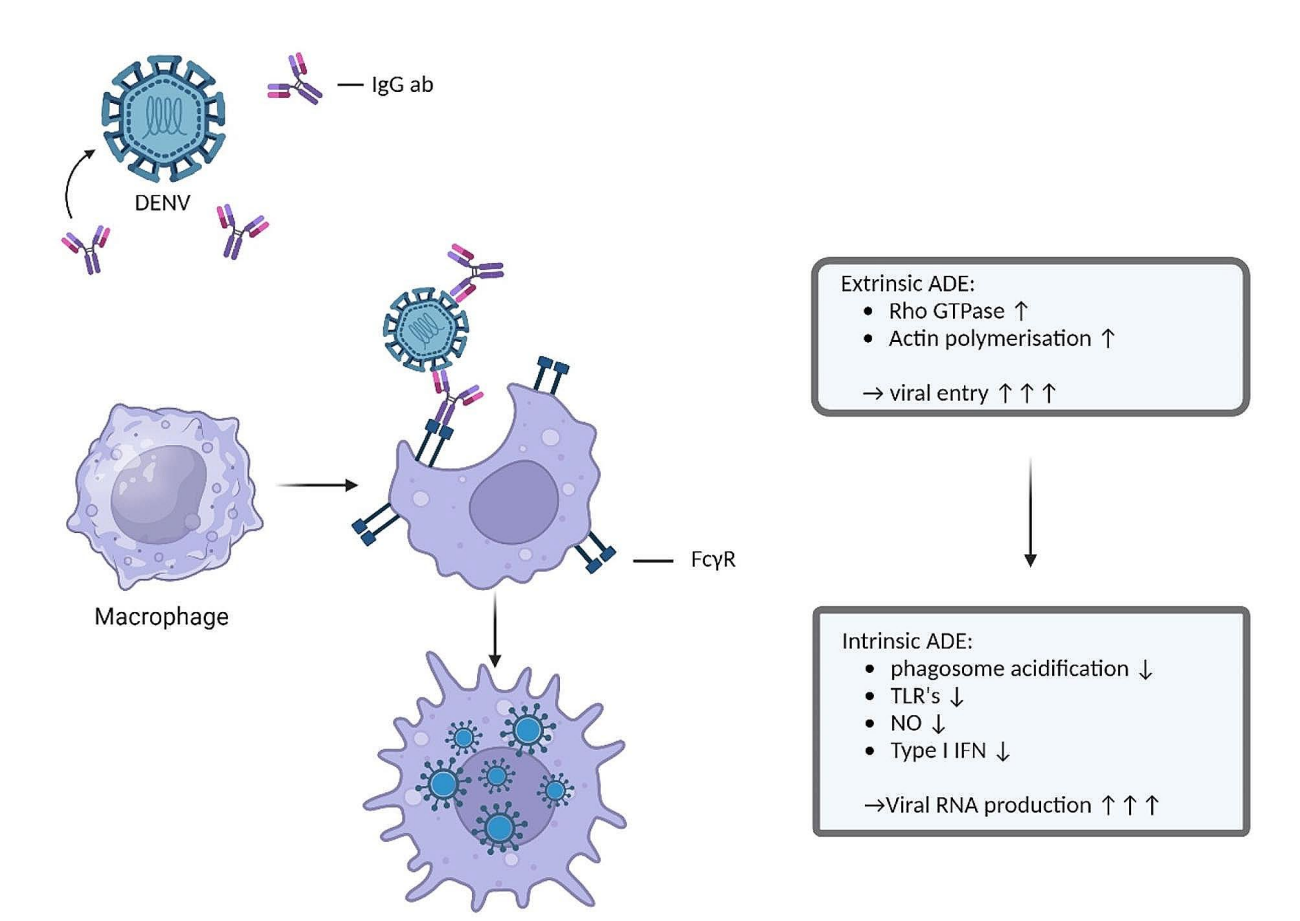
Schematic representation of Antibody-dependent enhancement in dengue infections. Antibody-dependent enhancement in dengue infections has both an extrinsic and an intrinsic component

Intrinsic ADE results in modulation of the immune system after the virus-antibody complex has entered the cells. The FcγRs will cluster and set off an intracellular signalling cascade resulting in activation of Rho GTPase and actin polymerisation. This will promote receptor internalisation and phagocytosis [54]. Once in the cell, the E protein undergoes conformational changes due to the lower pH intracellularly. This switch from a dimeric to a trimeric state provides fusion of viral and endosomal membranes, essential to establish release of the viral genome to the cytoplasm of a host cell [55–57]. Off note, E protein conformational changes is a mechanism seen in multiple viral infections, also to enhance viral entry, for example in HIV, the E protein changes conformation upon binding with an Ig and a CD4 receptor [58, 59]. DENV uses intrinsic ADE to modify the cellular and systemic immune reaction. This could be established by the weak binding between Ig and antigen. Antibody-opsonised DENV uses, after the immune complex dissociates, leukocyte immunoglobulin-like receptor-B1 (LILRB1) and SH2 domain-containing phosphatase-1 (SHP-1) to downregulate phagosome acidification and to escape lysosomal degradation [60]. The suppression of Toll-Like Receptors (TLR) has also been reported, resulting in suppression of pro-inflammatory cytokines and type I interferons [61, 62]. Additionally, increased productions of anti-inflammatory cytokine IL-10, together with high levels of IL-6 can inhibit Nitrous Oxide (NO) synthesis and decrease type I interferon production, resulting in an increase in viral RNA production [63]. This is achieved through suppression of the Janus kinase-signal transducer and activator of transcription (JAK-STAT) signalling pathway. Thereby the NO synthesis decreases together with inhibition of type I interferons which gives path to increased production of virions [62–64]. Intrinsic ADE mostly affects the innate immune response, with some effects on the adaptive immune system. In particular a shift to a T-helper-2 (Th2) biased immune reaction is established, promoting B-cell proliferation and thereby exacerbating the production of non-neutralizing antibodies [65]. An overview of the different mechanisms is given in Figs. 1 and 2.

### Vaccines

As has been discussed earlier, given the magnitude of dengue-related mortality and morbidity with dengue now emerging also in sub-tropical regions, a strong desire for an effective vaccine exists. In light of the scarce therapeutic options, vaccination is the obvious method to combat the millions of infections globally each year. However, the risk of triggering vaccine-enhanced disease or ADE poses a threat during the development of an effective and safe vaccine. Considering the challenges posed by disease-enhancing cross-reactive antibodies, the objective of vaccine developers was to develop a vaccine with protective antibodies against all DENV serotypes [66, 67].

**Fig. 3:**
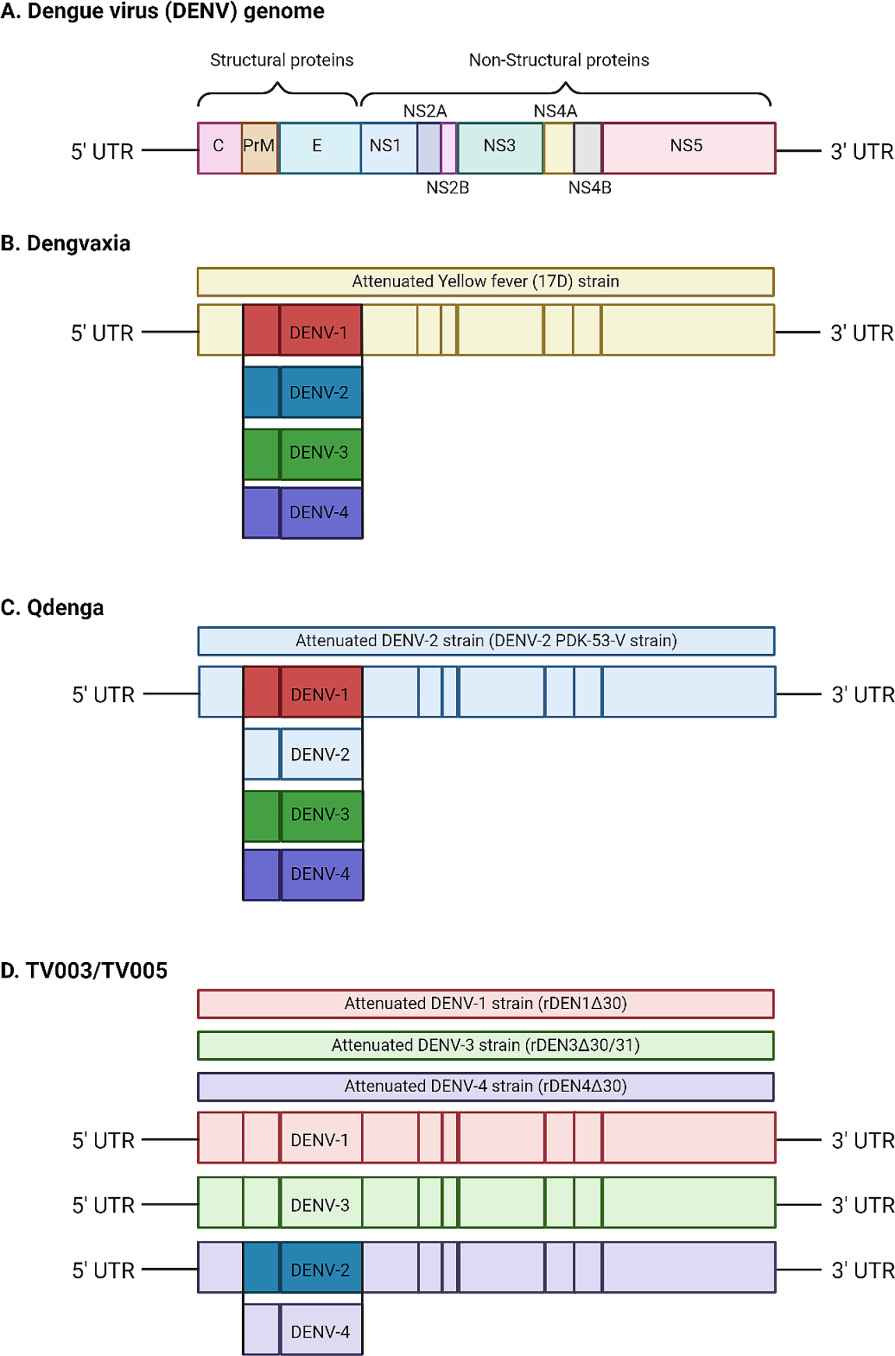
Schematic representation of the dengue virus **(A)**, the tetravalent vaccines Dengvaxia **(B)** and Qdenga **(C)** and the vaccine formulation TV003/TV005 **(D).** The dengue virus has an RNA genome with an open reading frame surrounded by untranslated regions. The open reading frame contains the genetic coding for three structural and seven non-structural (NS) proteins. The three structural proteins (capsid (C), premembrane (prM), and envelope)) form the structural components of the dengue virus particle

Dengvaxia (CYD-TDV, Sanofi Pasteur) is a tetravalent attenuated chimeric yellow fever vaccine introduced in 2015. It incorporates pre-membrane (prM) and envelope (E) genes from each DENV serotype into a backbone existing of non-structural (NS) genes of yellow-fever virus (Fig. 3B). Phase 1 and 2 studies demonstrated that a 3-dose regimen of CYD-TDV was well-tolerated in adult subjects [67, 68]. Despite the representation of all serotypes in the tetravalent vaccine, it predominantly elicited serotype-specific antibodies against DENV-4. Cross-reactive antibodies neutralized the remaining serotypes [69]. Therefore the vaccine efficacy also dropped after 2 years to 72,7%. Although the vaccine initially seemed to provide reasonable protection against dengue-related hospitalization after 2 years (89,2% overall and 72,6% in children of all ages), the risk of severe dengue was found to be higher in vaccinated children who were seronegative at baseline [4, 70]. This vaccine-enhanced disease was particularly evident in the younger age group (2–9 years), due to their lower probability of being exposed to DENV [68]. A possible cause of the increased amount of SD may be the usage of a yellow fever backbone, only incorporating 2 structural genes. NS-1 has been elicited to be a major pathogenic part of DENV by playing an important role in causing vascular permeability and plasma leakage in dengue infections [71]. NS1-specific antibodies could protect against these effects [72]. Due to the lack of non-structural genes of DENV in the vaccine of Sanofi Pasteur, vaccination with CYD-TDV does not result in the production of antibodies against dengue NS1, but instead to yellow fever NS1. These yellow fever NS1 antibodies might bind to dengue NS1 but not neutralize dengue NS1. Therefore, these yellow fever NS1 antigens hypothetically could play a role in ADE development or solely, due to the lack of a neutralizing antigen. This could result in a more severe disease presentation [73]. Consequently, to the increased risks of severe dengue and hospitalisation, adjustments were made to the license for the vaccine’s use. CYD-TDV is currently licensed as secondary prevention after laboratory-confirmed previous dengue infection for individuals aged 6–16 years (U.S. Food and Drug Administration (FDA)) and 6–45 years (European Medicines Agency (EMA)) or in areas with high (> 80%) seroprevalence [74–76]. As a result, the use of CYD-TDV is limited.

By the end of 2022, a second live attenuated vaccine targeting DENV obtained licensing under the name Qdenga (TAK-003, Takeda) and has since been registered in various regions, including the European Union, the United Kingdom, Brazil, Argentina, Indonesia, and Thailand [77]. The EMA has authorized the use of TAK-003 in both adults and children aged 4 and above, regardless of serostatus, thereby expanding its applications in travel medicine [67, 78]. Although vaccine-enhanced disease has not been demonstrated after receiving TAK-003, several European countries maintain a cautious policy, predominantly recommending the vaccine in cases of previous, laboratory-confirmed, dengue exposure [79–81]. TAK-003 is formulated based on a live-attenuated DENV-2 strain (PDK-53-V), supplemented by the prM and E genes from the four distinct DENV serotypes (Fig. 3C). In contrast to CYD-TDV, where immunodominance was observed for type 4, TAK-003 exhibits the highest efficacy against serotype 2 [82]. Similar to CYD-TDV, no non-structural proteins for all DENV serotypes are represented in the genomic sequence of Takeda’s vaccine. The genomic code has been used of the structural proteins prM and E. This could explain the higher efficacy against DENV-2, as the whole genome of this serotype is used. The overall efficacy one year after the 2-dose regimen was consistent between seronegative and seropositive individuals at baseline, hovering around 80%. [83] However, at 3 years post-vaccination, the overall efficacy against symptomatic dengue had declined to 62,0%, which remained stable with 61.2% efficacy at 4.5 years [82, 84]. Efficacy against hospitalization was 84,1% (85,9% for seropositive participants, and 79,3% for seronegative participants). Currently, studies are ongoing to investigate the effect of booster vaccination [85].

Multiple DENV vaccines candidates are still under development. The live attenuated tetravalent vaccine Butantan-Dengue Vaccine (Butantan-DV) is currently in a phase 3 study. This vaccine is analogous to the TV003 formulation created by the U.S. National Institute of Allergy and Infectious Diseases (NIAID) [86]. This formulation was developed by deleting 30 nucleotides in untranslated regions (UTRs) of DENV-1 (rDEN1Δ30), DENV-3 (rDEN3Δ30/31) and DENV-4 (rDEN4Δ30). A DENV-2 component was engineered as a chimeric virus by replacing the prM and E proteins of rDEN4Δ30 with those of DENV-2 (Fig. 3D) [68, 70, 81]. In a phase 3 study, two-year vaccine efficacy was 79,6% (89,2% for participants with previous dengue exposure and 73,6 for participants without) [86]. Efficacy was highest for DENV-1 at 89,5%, with efficacy for DENV-2 at 69,6%. No cases of DENV-3 or DENV-4 were observed during the study period. A cause for the lower efficacy against DENV-2 could be the formulation, with the DENV-2 component based on the DENV-4 virus. The incidence of dengue with warning signs or severe dengue was reportedly low, however no number was given [86]. An advantage of the Butantan vaccine is the need for only one vaccine dose, compared to TAK-003 where two doses are needed, three months apart. This makes vaccination in low-resource areas more accessible.

TV005 is another vaccine formulation created by the NIAID, similar to the TV003 formulation of the Butantan vaccine, however, it contains a 10-fold higher amount of the DENV-2 component. In a phase 2 trial, vaccination was well tolerated, with a rash as the most common side effect (26% for vaccinated recipients vs. 12% for placebo recipients) [87]. After vaccination 83% of participants were seropositive for DENV-1, 99% for DENV-2, 96% for DENV-3 and 87% for DENV-4. Antibody titres were higher for participants with previous dengue exposure than for participants without (10–15 fold for DENV1-3, 1,6 fold for DENV-4). After three years, most adults and adolescents remained seropositive for all serotypes, but seropositivity decreased in children. Two controlled human infection studies investigated the protection of TV005 against DENV-2 and DENV-3 [88]. Participants were challenged with either DENV-2 or DENV-3 6 months after being vaccinated with either TV005 or placebo. No vaccinated participants had viremia after being challenged with either DENV-2 or DENV-3, compared to all placebo recipients. Additional research is needed to determine vaccine protection over time, and protection from SD and hospitalization, but the results of these studies are promising for the development of TV005 as another dengue vaccine.

Several other vaccine candidates are currently in pre-clinical or phase I studies, representing a diverse array of vaccine types. These include inactivated virus vaccines, subunit vaccines, DNA vaccines, and viral vector vaccines [66]. As said before, both a tetravalent vaccine and the government of a serotype specific immune response are the two major requirements regarding vaccine development. Whole genome vaccines such as the above described live-attenuated vaccines have the disadvantage that antigenic parts of DENV, exhibiting highly immunogenic properties, are not necessarily serotype specific. Especially the fusion loop epitope on domain II of E and the prM induce the most heterotypic antibody’s cross reacting generally among DENV serotypes. These are also the most immunodominant epitopes of DENV [89]. The E protein as a whole contains three different domains heterogenic capabilities regarding immunogenicity and serospecificity. Domain three is the part with the most serotype specific genome, causing minimally cross-reacting immunoglobulins and therefore reducing the chance of ADE. [90–93]. Yet, the difficulty is that only a small part of the Igs are produced against domain III. Much higher percentages are, as explained earlier, directed against prM protein or the fusion loop of domain II of E protein, due to their immunodominance [94]. In other words, maintaining epitopes of domain II in the genomic code of vaccines could be a remaining source of the possibility of ADE development. Therefore, subunit or multi-epitope vaccines could be a solution, due to their feasible to only implement serotype specific epitopes, which do not induce cross-reactivity. Immunoglobulins against DENV mostly recognize structural proteins with immunogenic potent epitopes. Yet, using small fractions of viral genomes as the base for a vaccine has the disadvantage that larger epitopes that could be recognized as a whole, are no part of the constitution of the antibody repertoire generated by the immune system as a reaction to this vaccine. This so cold, quaternary epitopes are also novel found sights of potentially inducing serotype specific antibodies. This is due to the capability of Igs to bind to a specific structure of the virus itself rather than to a specific epitope. These are Igs which are referred to as quaternary structure specific since they exclusively recognize a unique structural piece of the E-protein [95]. Regarding domain III- E protein-based subunit vaccines, recent research has shown that quaternary epitopes induce a potent immune reaction [91].In clinical trials testing both subunit vaccines based on small peptides or larger quaternary epitopes no grand success is yet achieved. Epitopes necessarily need to activate induction of potent Igs without the other structural immunodominant incorporated in the genomic code of a vaccine. Therefore, adjuvants and more knowledge about the right components necessary are needed [96].

Another example could be given by not looking only at the humoral response, but also at the most potent inducers of cellular immunity. Non-structural proteins are mostly the favored epitopes regarding T-cells. CYD-TDV did only induce a humoral response, without inducing cellular immunity due to the lack of non-structural proteins of DENV. CD4 + T cells recognize epitopes that can be found on structural proteins E and capsid, as well as the non-structural NS1 protein, in an HLA-dependent manner [97]. The NS5 protein was identified as exhibiting the most conserved epitopes across the serotypes, which are likely to be crucial for viral replication. NS3 protein has been identified as the most immunogenic antigen for cellular response against DENV and has multiple conserved genomic regions which are highly preserved among the serotypes and also with other Flaviviruses. Thus, the human CD8 + T cell responses induced by a live attenuated tetravalent dengue vaccine are directed against highly conserved epitopes, among DENV virus and even across the different Flaviviridae [98]. As said earlier, therefore incorporating NS-1 of YFD or the DENV serotypes will maintain the possibility of the generation of cross reactive Igs [73, 99]. Therefore, also vaccines only inducing a cellular response, without inducing a humoral response are being development and tested in clinical trials [100].

Next to the great challenge of vaccine development, the potential success of a vaccine is also dependent on the implementation into public health and vaccination programs. This, to achieve acceptance of a vaccine and to ensure adequate coverage rates, culminating in maximizing the public health benefit. Studies on potential vaccine acceptance have highlighted a broad-spectrum interest in a dengue vaccine [101, 102]. When looking at vaccine implementation in general, due to the unknown level of adherence and acceptation when largely implemented, hesitance among public health policy makers governing new vaccines is seen. Introductions of new vaccines in the last decades, such as the HPV vaccine, have faced challenges in uptake due to factors related to the specific indications, reaching the target population, disease awareness and various programmatic and societal difficulties, which have impeded early vaccine coverage [103, 104]. Related to dengue vaccines, implementation into public health initiatives has so far been the case in Brazil and the Philippines [105]. In Brazil for example, the state of Paraná decided to implement CYD-TDV without any cost for recipients, in the battle against the high number of DENV related infections in the region between 2016 and 2018. This resulted in a vaccine coverage rate above 60% for the first dosage. Yet, in 2018 during the campaign, as explained earlier, due to the increased risks of severe dengue and hospitalization among seronegatives, adjustments were made to the license for the vaccine’s use. This could explain the lowering vaccine coverage rate seen in Paraná during the second and third vaccination, respectively 44.2% and 28.6% [106, 107]. A recent study investigating the effectiveness of the CYD-TDV vaccination strategy in Paraná, showed that vaccination in individuals seropositive at baseline effectively reduced the incidence of dengue. In individuals seronegative at baseline however, vaccination was not associated with diminution of the overall chance of a dengue infection. This was due to a higher incidence of DENV-2 related cases [108]. This study’s results are in line with the manufacturer’s findings regarding outcomes of the vaccine and thereby the study confirmed as one of the first that indeed implementation of CYD-TDV should be only in a very narrowed target population [109]. Data regarding vaccine efficacy after broad implementation have not been published for the Philippines. Hitherto, no other countries have elicited a large vaccine campaign or vaccination strategy. With the arrival of TAK-003, despite no evident signs of ADE, after 4,5 years of follow-up, no initiatives have been elicited to apply the vaccine on a broad scale as well. Nevertheless, not sufficient clinical data has been gathered regarding TAK-003’s safety in serotypes DENV-3 and DENV4. In more detail, when looking at the data, seronegatives were more often suffering a symptomatic DENV-3 infection or in need of hospitalization due to DENV-3. This surplus was minor and statistically not reaching significance [82]. Acknowledging the experiences with CYD-TDV, the above-mentioned studies nevertheless enlighten the difficult subject of implementation of a vaccine suffering safety issues when applied on a broad scale within a heterogenous population. The efficacy shown of both CYD-TDV and TAK-003 in highly endemic countries could be of great significance preventing SD, but nevertheless multiple of the above-mentioned factors could contribute to potential hinders to achieve implementation on a big scale. A possible solution could be pre-vaccination screening. Yet, WHO’s Strategic Advisory Group of Experts on Immunization (SAGE) addressed that TAK-003 could be used without pre-vaccination screening despite the insufficient amount of data regarding DENV-3 and DENV-4. This consideration is primarily based on the grand reduction of impact on public health, taking into account that seronegatives have a proven significantly reduced risk of DENV related infections by two of the four serotypes [82, 84, 110]. Notwithstanding high-income countries, where TAK-003 could be of importance in travel medicine. Yet, the use of this vaccine in travelers living in non-endemic countries requires much more consideration. Examples of possible hiccups, regarding travelers specifically, that are added to the scope of remaining uncertainties, are the waning levels of Igs when not being exposed to DENV and their neutralizing capability after several years without exposure to DENV. The latter has not been fully elicited, and could possibly be a not yet identified source of ADE [82, 84].

## Conclusion

Antibody-dependent enhancement is a phenomenon causing worldwide morbidity and mortality in dengue virus infections. Additionally, in the search for a vaccine against DENV, vaccine-induced ADE has caused several setbacks for the use of a vaccine on a larger scale. Despite this, a recently licensed live attenuated vaccine (Qdenga, TAK-003, Takeda) has not shown signs of ADE. Long-term data has yet to show whether new candidates in phase 2 and 3 trials such as TV003/TV005 with both structural as well as non-structural proteins have a higher efficacy. Another big question remaining is if vaccine efficacy is waning, could that result in more ADE?

## Data Availability

No datasets were generated or analysed during the current study.
